# Effect of Exosomes from Rat Adipose-Derived Mesenchymal Stem Cells on Neurite Outgrowth and Sciatic Nerve Regeneration After Crush Injury

**DOI:** 10.1007/s12035-018-1172-z

**Published:** 2018-06-21

**Authors:** Vesna Bucan, Desiree Vaslaitis, Claas-Tido Peck, Sarah Strauß, Peter M. Vogt, Christine Radtke

**Affiliations:** 1 Department of Plastic, Hand and Reconstructive Surgery, Hannover Medical School, Carl-Neuberg-Strasse 1, 30625 Hannover, Germany; 2 Department of Plastic, Hand and Reconstructive Surgery, Hannover Medical School, Feodor-Lynen Str. 21, Hannover, Germany; 3 Department of Plastic and Reconstructive Surgery, Medical University of Vienna, Währinger Gürtel 18-20, 1090 Vienna, Austria

**Keywords:** Microvesicles, Neurites stimulation, Axons regeneration, adMSCs

## Abstract

Peripheral nerve injury requires optimal conditions in both macro-environment and microenvironment for promotion of axonal regeneration. However, most repair strategies of traumatic peripheral nerve injury often lead to dissatisfying results in clinical outcome. Though various strategies have been carried out to improve the macro-environment, the underlying molecular mechanism of axon regeneration in the microenvironment provided by nerve conduit remains unclear. In this study, we evaluate the effects of from adipose-derived mesenchymal stem cells (adMSCs) originating exosomes with respect to sciatic nerve regeneration and neurite growth. Molecular and immunohistochemical techniques were used to investigate the presence of characteristic exosome markers. A co-culture system was established to determine the effect of exosomes on neurite elongation in vitro. The in vivo walking behaviour of rats was evaluated by footprint analysis, and the nerve regeneration was assessed by immunocytochemistry. adMSCs secrete nano-vesicles known as exosomes, which increase neurite outgrowth in vitro and enhance regeneration after sciatic nerve injury in vivo. Furthermore, we showed the presence of neural growth factors transcripts in adMSC exosomes for the first time. Our results demonstrate that exosomes, constitutively produced by adMSCs, are involved in peripheral nerve regeneration and have the potential to be utilised as a therapeutic tool for effective tissue-engineered nerves.

## Introduction

Numerous surgical procedures are performed to repair peripheral nerve injuries. Nerve lesions defect with a short gap are usually treated by end-to-end anastomosis. Traumatic injuries resulting in longer peripheral nerve lesions often require a graft to bridge the gap. Although autologous nerve autograft is still the first-choice strategy in reconstructions, they have several disadvantages caused by the limited availability of donor tissue, sacrifice of functional nerve, and potential formation of neuroma [1–4]. The development of new therapeutic strategies to improve and especially accelerate axonal nerve regeneration is of great importance.

Many experimental studies have been conducted to find alternative conduits, using various synthetic or biological substances [5–8]. The cell-based therapies with autologous Schwann cells (SCs) play a pivotal role in peripheral nerve regeneration. When seeded in artificial nerve conduits, Schwann cells have been shown to enhance nerve regeneration [9–11]. Furthermore, proliferating Schwann cells release neurotrophic factors [12] and form the bands of Büngner to direct regenerating axons across the lesion. However, the clinical benefits of SCs are limited by their inability to generate sufficient cell numbers quickly. Stem cells are an attractive cell source for regeneration and repair processes of injured tissue as they are able to self-renew with a high growth rate and possess multi-potent differentiation properties. Recent studies demonstrate the effect of mesenchymal stem cells (MSCs) for enhancing tissue regeneration [13, 14] and show that adMSCs have a pro-regenerative phenotype when following transplantation into the injured peripheral nervous system. These cells secrete neurotrophic factors [15, 16], recruit Schwann cells to aid the regenerative process, and enhance the survival of sensory and motor neurons [17]. Nonetheless, the effect of stem cells in tissue regeneration depends primarily on their capacity to secrete soluble factors, chemokines, cytokines or growth factors, creating optimal environmental conditions for tissue regeneration [18–22].

In addition to soluble factors, MSCs are known to secrete extracellular vesicles which are involved in cell-to-cell communication and have a regenerative effect on the heart, kidney, liver, and nervous tissue [23–26]. The exosomes constitute one subtype of secreted microvesicles (MVs). They emerge through inward budding of so-called multi-vesicular endosomes (MVE), and their membrane is enriched in certain lipids [27, 28]. Their function was examined in several studies, but their mechanism of action remains relatively unclear [29, 30]. Some studies have shown that MVs contribute to improved recovery from acute kidney injury of mice and accelerated liver regeneration of hepatectomised rats [31, 32]. Furthermore, they efficiently replace the MSC exosomes in reducing the infarct size in myocardial ischemia [33]. These microvesicles have been tested in preclinical settings for the treatment of neurological diseases [34]. The exosomes secreted by Schwann cells increase neurite growth substantially and enhance axonal regeneration [35]. Ferinazzo et al. show the relevance of adMSC vesicles as a source of remyelinating and regenerative factors, which might modulate the microenvironment in neuroinflammatory as well as in neurodegenerative disorders [36]. The use of MVs reduces the risks of dysfunction and transformation of transplanted stem cells and can be used as an alternative non-cell-based therapy for tissue regeneration [37, 38].

In the present study, we examine the influence of isolated adMSC exosomes on neurite outgrowth of DRG (dorsal root ganglion) neurons in vitro and analyse the effect with regard to peripheral nerve regeneration after sciatic nerve injury in a rodent model.

## Materials and Method

### Animals

Adult male female Crl:WI (Wistar) rats, 7–9 weeks old, weighing 350–400 g (*n* = 12) (Charles River, Sulzfeld, Germany) were used in the rodent model. The animals were housed under standard conditions, and the experiments were carried out in accordance with the guidelines of the German Animal Welfare Act (TV-Nr.14/1407).

### Adipose-Derived Mesenchymal Stem Cell Harvest and Cell Culture

The fat pads were carefully dissected from rats under inhalatory isoflurane anaesthesia; afterward, animals were sacrificed. After rinsing with Hank’s balanced salt solution (HBSS; PAA, Cölbe, Germany) and mincing, the fat tissue was digested with collagenase type I, CLS I (2 mg/ml; Biochrom, Berlin, Germany) for 60 min at 37 °C under shaking. After centrifugation step for 10 min at 620×*g*, the cell pellet was resuspended in adMSC culture medium: DMEM/F12 (Biochrom) with 100 U/ml penicillin, 100 mg/ml streptomycin (P/S; PAA), 0.2 mM l-ascorbic acid-2-phosphate (A2P; Sigma), and 10% foetal bovine serum (FBS; Biochrom). Cells were maintained at 37 °C, 100% air humidity, and 5% CO_2_.

### Isolation of the Exosomes from adMSCs

Cell culture media was harvested from adMSCs, grown initially in the presence of 10% foetal bovine serum (FBS) and without FBS for the last 12 h. After 12 h, cells and debris were removed by centrifugation at 350×*g* for 10 min and 2000×*g* for 30 min. For ultracentrifugation, the samples were pre-enriched at 100,000×*g* for 70 min at 4 °C using a Ti45 rotor (Beckman) and Beckman ultracentrifugation tubes (355622) on a Beckman Optima XPN-100. For precipitation, Total Exosome Isolation kit (Thermo Fisher Scientific, USA) was added to the cell and debris-free cell medium (1:2 with exosome isolation reagent and cell medium, respectively). Cell medium and the exosome isolation reagent were mixed by brief vortexing and incubated at 4 °C overnight before centrifugated at 4 °C at 10,000×*g* for 1 h. The pellet containing pre-enriched exosomes was resuspended in PBS [39].

### PKH-26 Staining of the Exosomes

For the detection of the exosomes in the acceptor cells, the vesicles were stained with PKH-26 (Sigma; St. Louis, USA) according to the manufacturer’s instructions. The adMSCs and Schwann cells were then cultured with labelled exosomes for 24, 48 and 72 h. After that, the cells were analysed using the ZEISS Axiovert 200 M fluorescence microscope [40].

### Western Blot Analysis

Fifteen micrograms of exosomes protein was fractionated by 15% SDS-PAGE and transferred to polyvinylidene fluoride membranes (Millipore Corporation, USA) and then blocked in Odyssey buffer for 1 h. Exosome marker expression levels were determined by immunoblotting with the following polyclonal antibodies: monoclonal anti-CD9 and anti-CD63 (Abcam; UK (1:500)), overnight at 4 °C. For quantification of protein expression levels, Odyssey 680/800 nm secondary conjugates (Li-Cor BioSciences, USA (1:2000)) were used and membranes were analysed using the Odyssey Infra-Red Imaging System and software (Li-Cor BioSciences, USA).

### Immunofluorescence

Exosomes and Schwann cells were fixed in 4% paraformaldehyde for 20 min and incubated with monoclonal anti-CD9 and anti-CD63 (Abcam; UK (1:100 dilution)) and Ki67 (Thermo Scientific, USA (1:100 dilution)) antibodies at 37 °C for 1 h, washed three times in ice-cold PBS and incubated with Alexa Fluor conjugated secondary antibody (Invitrogen, USA (1:600 dilution)) at 37 °C for 30 min. After three washing steps with PBS, sample were dried and covered with Immunomount medium (Thermo Scientific; USA). Images were acquired using ZEISS Axiovert 200 M fluorescence microscope equipped with the appropriate barrier filters.

### Flow Cytometry

Exosomes (10 μg of protein) were bound to 5 μg aldehyde surface latex beads (Invitrogen) for 1 h at room temperature. Bound exosomes were spun down and incubated in FACS permeabilization buffer. The unoccupied sites were saturated with vesicle free foetal calf serum, and the exosomes were incubated with primary antibodies anti-CD9 and anti-CD63 or control isotype for 1 h at room temperature. The exosomes were spun down and incubated with FITC-conjugated secondary antibodies for 30 min at room temperature. The staining was analyzed by FITC fluorescence detection using a FACScalibur (Becton Dickinson).

### Immunohistochemistry

Sciatic nerves from transplanted and control rats were processed for immunocytochemistry as described previously. Sciatic nerves were removed and postfixed for 20 min in 4% paraformaldehyde. Tissue was then cryoprotected in 30% sucrose in 0.14 M Sorensen’s phosphate buffer overnight at 4 °C. Ten-micrometer longitudinal cryosections of the sciatic nerves were cut and mounted on Silane Prep glass slides (Sigma, St. Louis, MO, USA). Sections were processed for immunostaining for monoclonal antibody neurofilament (NF, Sigma, St. Louis, MO, USA; dilution 1:1000) followed by incubation with secondary antibody goat anti-mouse IgG-Alexa Fluor 594 (Invitrogen, Eugene, OR, USA; 1:1000) and coverslipped with DAPI-containing mounting media (VectaShield, Vector Laboratories, Burlingame, CA, USA). The sections were examined with a fluorescence microscope (Nikon Eclipse 800; Spot RT Colour CCD camera; Diagnostic Instruments).

### Isolation of Schwann Cells from Rodents

Adult male female Wistar rats were anaesthetised and sacrificed. The sciatic nerve was removed on both sides and transferred to a sterile work bench. The removal of epineurium and connective tissue was performed on ice. The nerves were kept in Hanks BSS (HBSS) + 1% (PAA Cölbe, Germany) Penicillin/Streptomycin (PAA, Cölbe, Germany) during preparation. Nerve fascicles were easily removed by pulling them out with sterile forceps. The fascicles were then cut into 3–4 mm pieces and transferred in 6-well dish (TPP, Trasadingen, Switzerland), followed by an incubation at 37 °C and 5% CO_2_ with DMEM high glucose (Biochrome, Berlin, Germany), + 10% FCS (Biochrome, Berlin, Germany), + 1% P/S (PAA, Cölbe, Germany) and + 1% sodium pyruvate (Biochrome, Berlin, Germany) for 3 weeks. After 7 days, first migration of cells was observable [41].

### DRG Harvest and Culture

Animals were sacrificed under isoflurane anaesthesia, ganglia were excised, washed in HBSS and then incubated in HBSS containing 1.7 mg/ml collagenase A (Roche, Germany), 1.7 mg/ml collagenase D (Roche) and 1.25 mM calcium chloride. After centrifugation, ganglia were incubated in PBS (Gibco) containing 2.5 mg/ml papain, 100 mM l-cystein and 10 mM EDTA (Sigma). The digested ganglia were gently dissociated in DMEM/F12 + 6% d-glucose (Sigma), then centrifuged and washed with PBS. The neurons at the base were resuspended in a modified Bottenstein and Sato medium (DMEM/F12 + 6% d-glucose supplemented with 100 μg/ml BSA, 100 μg/ml transferrin, 100 μM putrescine, 30 nM sodium selenite, 20 nM progesterone, 10 nM insulin and 1% P/S (Sigma)), counted and plated onto laminin-coated 12 mm glass coverslips in 24-well plates at 1000 neurons/coverslip.

### DRG Neuron and Exosomes Co-culture

The DRG neurons were cultured with and without exosomes in normal DRG medium (‘DRG only’) and in co-culture with adMSCs. After 24 or 48 h, cultures were washed with PBS and fixed in 4% PFA. Phase contrast images (2080 × 1544 pixels, 2.9 pixels/μm) of all neurons were taken at 10-fold objective magnification on CKX41 imaging system (Olympus, Germany). Neurons were analysed morphometrically using ImageJ (National Institute of Health, USA) software.

### RT^2^ Profiler™ PCR Array

To analyse the presence of neural growth factors in adMSC exosomes, the RT2 ProfilerTM PCR Array System (Qiagen, Germany) was used according to the manufacturer’s instructions. The three steps of the cycling program were 95 °C for 10 min for 1 cycle, then 95 °C for 15 s, 55 °C for 40 s and 72 °C for 30 s and repeated for 40 cycles using Bio-Rad iCycler (Bio-Rad, USA). All reactions were performed with the SsoFast EvaGreen Supermix (Bio-Rad, USA) in a total volume of 15 μL. The relative expression intensity was obtained by calculating the 2^−△Ct^ for each sample.

### Nerve Crush Injury and Exosomes Implantation in the Sciatic Nerve

For exosome implantation, adult male female Crl:WI (Wistar) rats were anaesthetised and the sciatic nerve was exposed by spreading the gluteal muscles apart. At the level of piriformis tendon, the nerve was tightly crushed with No. 5 Dumont forceps for 10 s until its colour became translucent. This procedure leads to complete transection of the axons in sciatic nerve but leaves the epineurium intact. After further steps (see implantation below), the wound was closed by suture. The PKH-26-stained adMSC exosomes were injected proximal and distal of the crushed nerve site with Hamilton syringe. After the operation, a weekly footprint analysis was performed for duration of 3 weeks. Finally, all animals were sacrificed and the sciatic nerves were harvested for further analysis.

### Footprint Analysis

Animals were tested preoperatively, 1-day postoperatively and then in weekly intervals. At days 7, 14 and 21 after the treatment, the rats were held at their chest and their hind limbs were dyed with stamping ink. After some conditioning trials, the rodents walked steadily through a tunnel with a dark shelter at the end leaving their marked footprints on a paper strip. A minimum of three footprint pairs were analyzed comparing the control to the experimental side. Prints were measured for the following parameters: distance between foot prints (TOF), the entire plantar length (PL), the distance from the first to fifth toes, the toe spread (TS), the distance between the second and fourth toes and the intermediary toe spread (IT). The Sciatic Function Index (SFI) was calculated according to the following formula:

$$ \mathrm{SFI}=\left(\left(\frac{\left(\mathrm{ETOF}-\mathrm{NTOF}\right)}{\mathrm{NTOF}}\right)+\left(\frac{\left(\mathrm{NPL}-\mathrm{EPL}\right)}{\mathrm{EPL}}\right)+\left(\frac{\left(\mathrm{ETS}-\mathrm{NTS}\right)}{\mathrm{NTS}}\right)+\left(\frac{\left(\mathrm{EIT}-\mathrm{NIT}\right)}{\mathrm{NIT}}\right)\times \frac{220}{4}\right) $$

The calculated index gives information on the functional condition of the peripheral nerve. A score of − 100% represents the total loss of function whereas 0% indicates an efficient nerve.

## Results

### Characterization of adMSC Exosomes

**Fig. 1:**
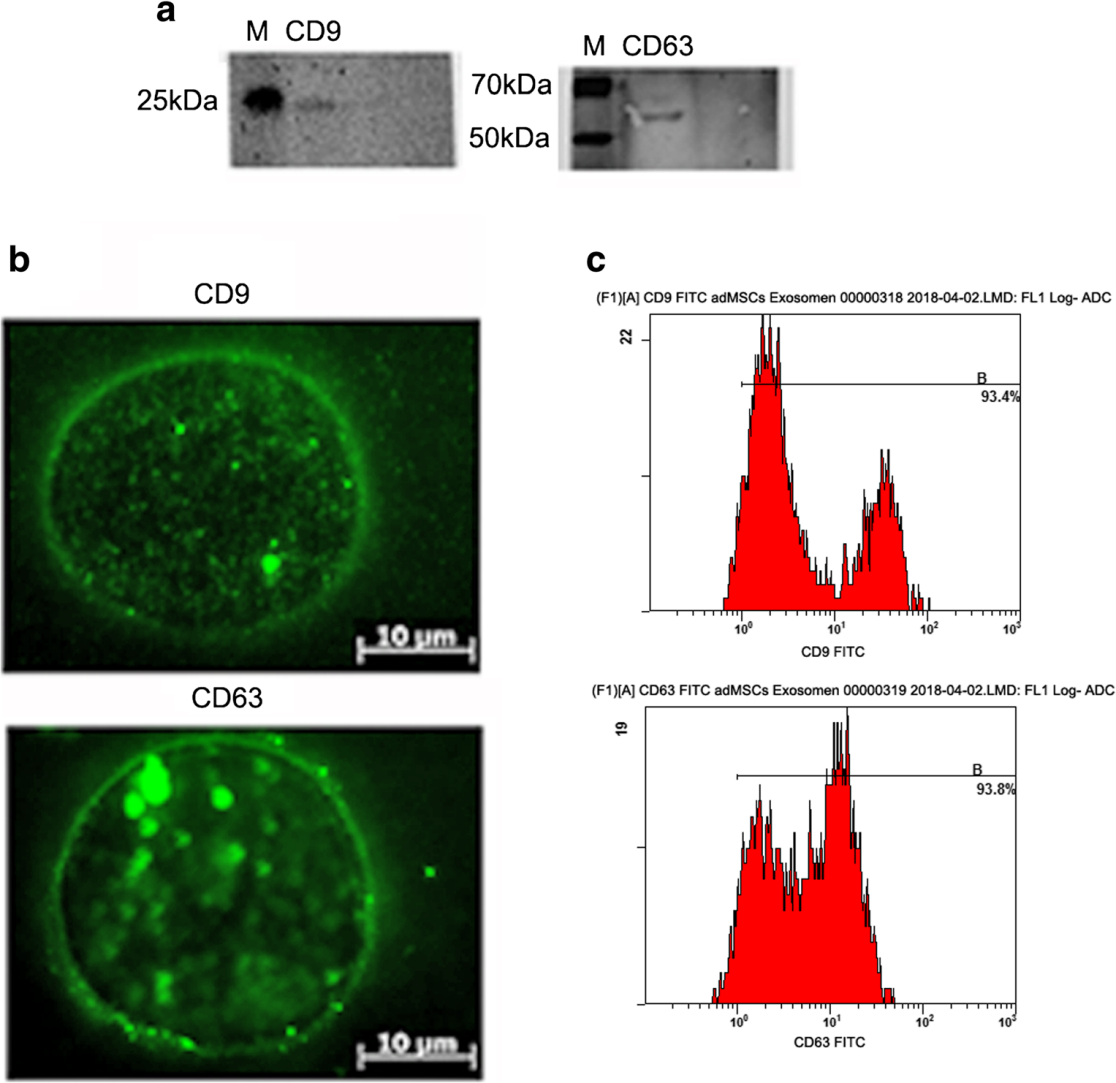
Exosome populations. **a** Total exosome isolation preparations were characterised by western blot. **b** immunofluorescence analysis. The blots were labelled for typical exosomal markers CD9 and CD63. M marker. **c** Flow cytometry analysis of exosomes collected from adMSC cells

Exosomes were isolated from the culture supernatants of rat adipose-derived MSCs by centrifugation. It is well described that exosomes contain various proteins, especially those of the tetraspanin family [42, 43]. Tetraspanins CD63 and CD9, the protein markers of exosomes, were detectable in the exosomes but absent in control PBS used to suspend the exosomes (Fig. 1a).

This result was supported by the immunofluorescence analysis of the CD63 and CD9 surface markers. For immunofluorescence analysis, exosomes were fixed and stained with mouse MAbs against the tetraspanin CD63 or CD9, followed by Alexa Fluor 594 anti-mouse (green). Single fluorescence sections are shown (Fig. 1b).

Collectively, these results reveal that rat adMSCs secrete exosomes to the culture supernatant and can be successfully isolated for further application.

### adMSC Exosomes and Schwann Cells

**Fig. 2:**
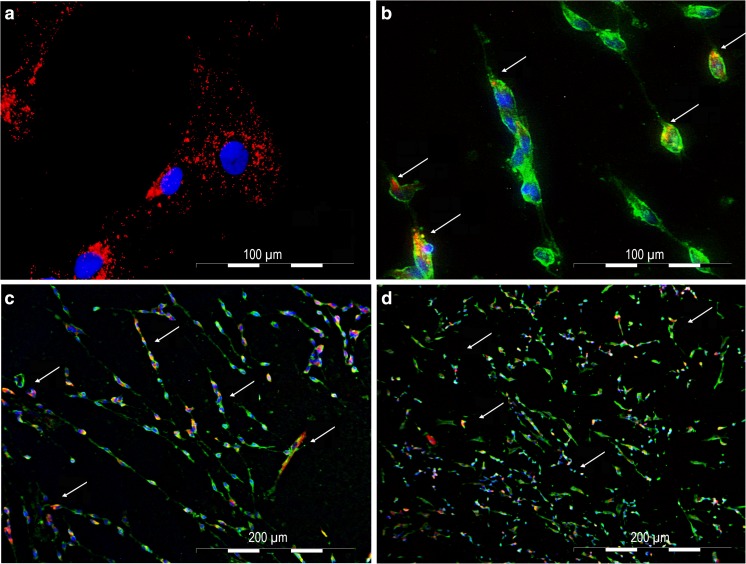
Exosome fusion. **a**, **b** Fusion of PKH-26-labelled exosomes with adMSCs and Schwann cells. adMSC exosome detection in Schwann cells after **c** 14 days and **d** 21 days

**Fig. 3:**
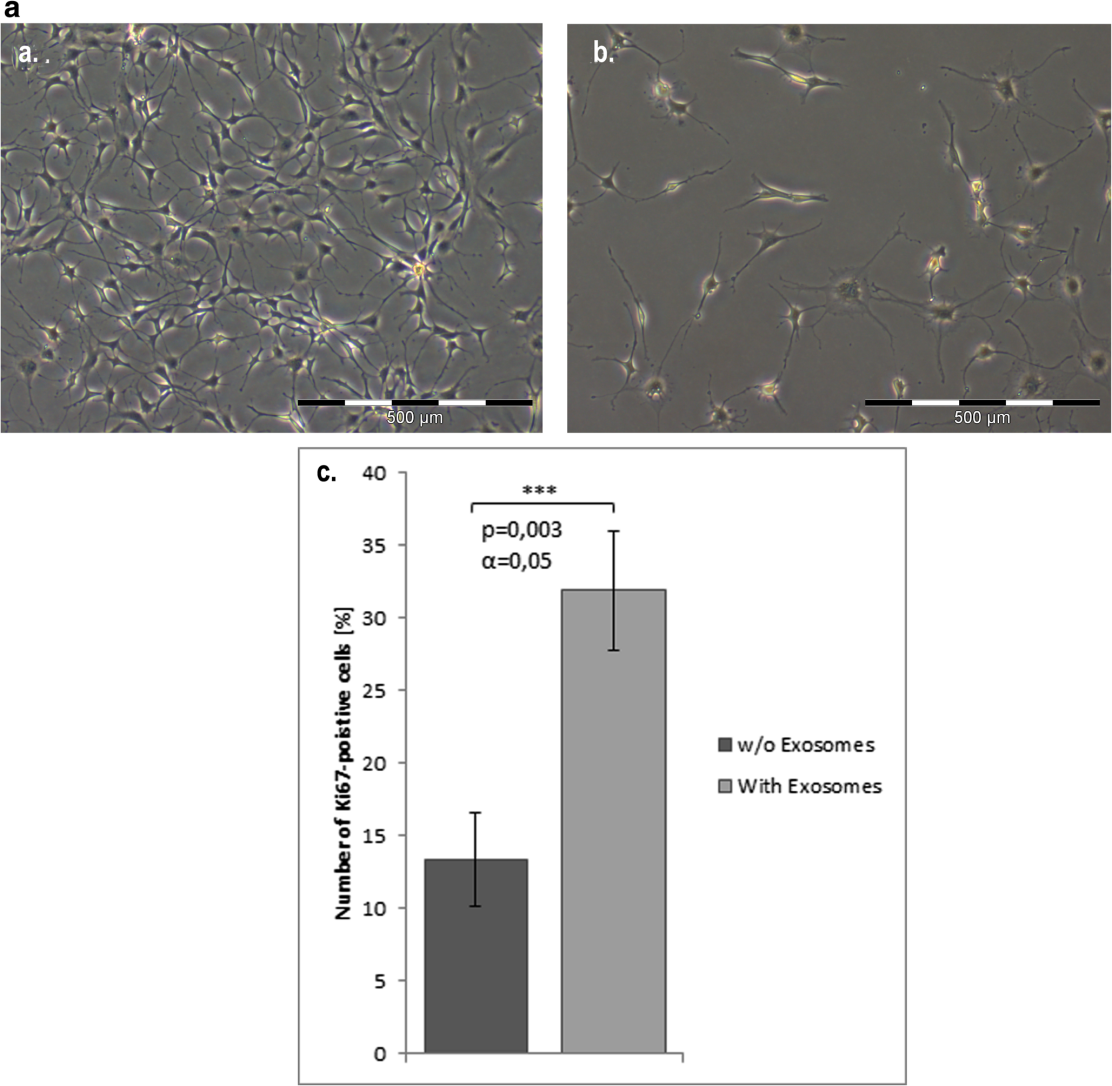
Schwann cell proliferation*.* **a** Morphologic characterization of Schwann cells proliferation, 4 days after stimulation with adMSC exosomes. **b** Schwann cells without exosomes. **c** Analyse the number of Ki67-positive cells

**Fig. 4:**
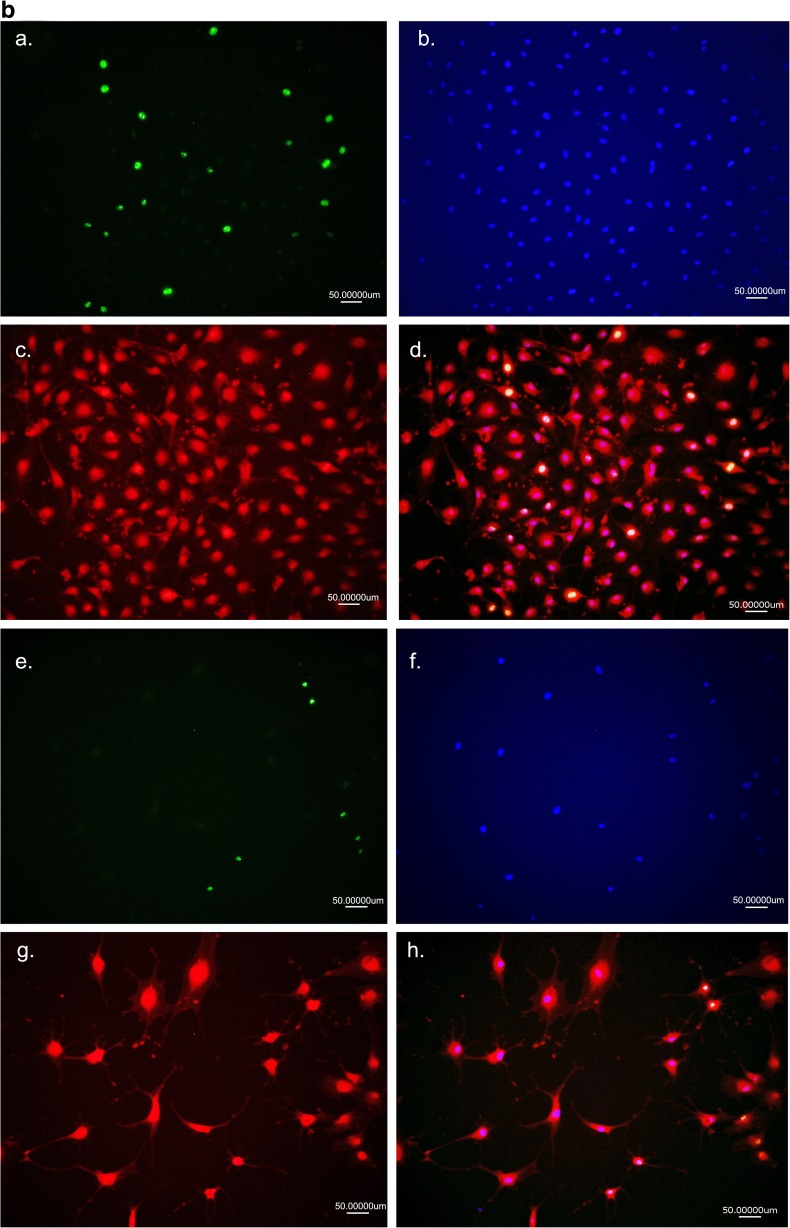
Detection of Ki67 protein, a nuclear protein that associated with cellular proliferation, in Schwann cells **a–d** with exosomes and **e–h** without exosomes. **a**, **e** Detection of Ki67 protein. **b**, **f** Detection of DAPI. **c**, **g** Detection of S-100 protein. **d**, **h** Overlap

In the next step, we examined the fusion of exosomes with adMSCs and Schwann cells. For that purpose, adMSC isolated exosomes were labelled with PKH-26 (Fig. 2a) and subsequently added to subconfluent adMSCs and Schwann cells culture. After overnight incubation with marked exosomes, numerous cells acquired positive PKH-26 signal (Fig. 2a, b), thus indicating that adMSC exosomes and their cargo can be transferred to the Schwann cells (Fig. 2b). Furthermore, after 14 and 21 days, we could prove the retrieval of the labelled exosomes in the Schwann cells culture (Fig. 2c, d). In addition, we showed that adMSC exosomes promoted the proliferation of Schwann cells 4 days after incubation (Fig. 3a) compared to the control (Fig. 3b). Also, the staining with Ki67 showed an increase interphase in Schwann cell nuclei treated with adMSC exosomes (Fig. 4a–d) compared to the controls (Fig. 4e–h). To analyse the number of Ki67-positive cells, Schwann cells without (*n* = 419) and with exosomes (*n* = 401) were counted. Here, the Schwann cells without exosomes are 13.37 ± 3.92% positive, whereas Schwann cells with exosomes achieve a significantly higher percentage of 31.92 ± 5.08% (Fig. 3c). Means and standard deviations were calculated and tested for statistical significance with Microsoft Excel using the paired *T* test.

### Neurite Outgrowth in Co-culture with Exosomes

**Fig. 5:**
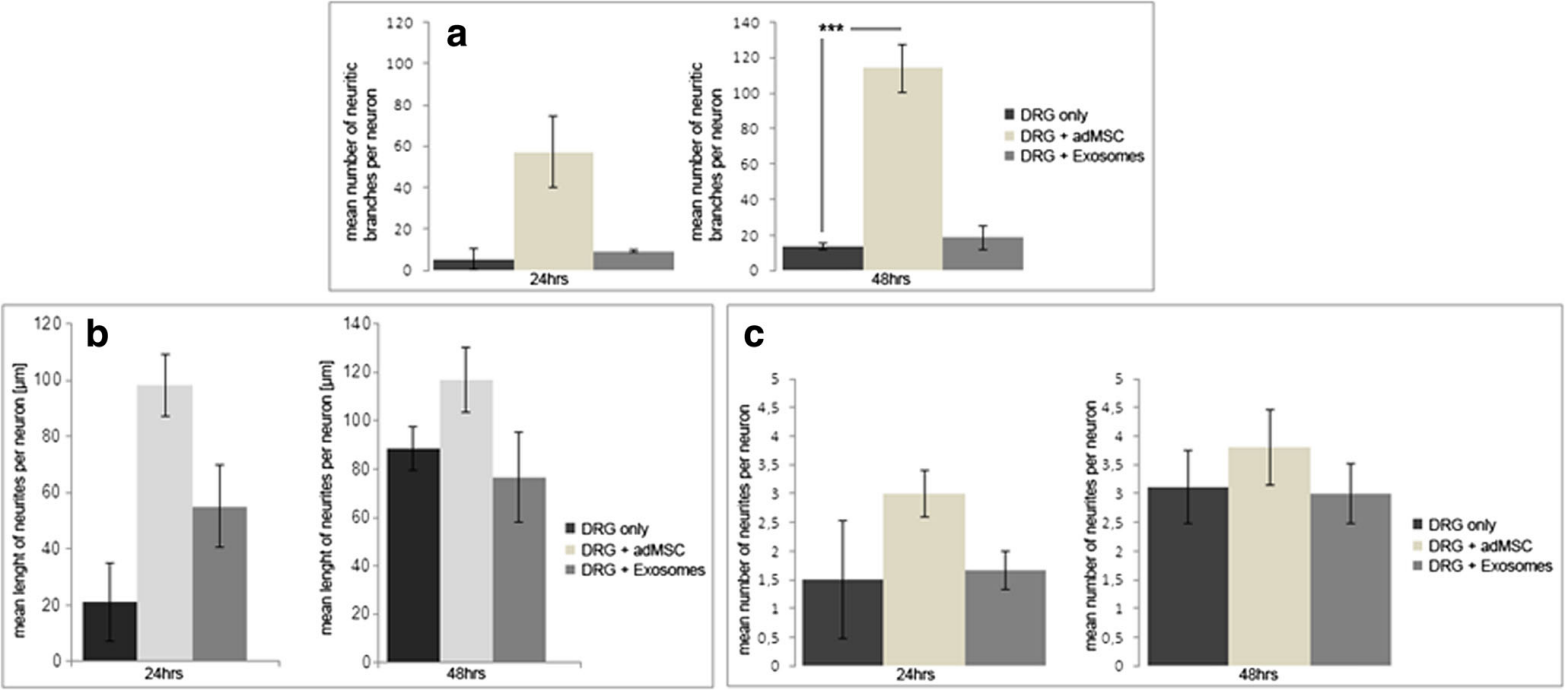
The neurotrophic effect of exosomes on DRG neurite outgrowth. The neurons from co-cultures of DRG neurons with adMSC exosomes were compared to DRG neurons in normal medium and with DRG neurons with adMSCs as positive control. Total lengths of neurites per neuron were determined. Data are given as mean plus standard deviation

To assess the effect of adMSC-derived exosomes on neuritic outgrowth, the isolated adMSC exosomes were cultured with rat primary DRG neurons for 24 and 48 h. Apart from the experimental group, consisting of the co-cultured cells, we used a DRG neuron-only culture (DRG) as negative control group and undifferentiated adMSCs as positive control group for co-cultivation with freshly dissociated DRG neurons. To quantify the effect of adMSC exosomes on DRG neurons, we studied the neurite length, neurites number and number of branching per neuron (Fig. 5). After 24 h, we detected 30.2 ± 27.1 μm of neurite length in single culture and 55.3 ± 28.7 μm neurite length in co-cultures with adMSC exosomes. Twenty-four hours later, the mean lengths in exosomes co-culture were 72.9 ± 26.4 and 88.4 ± 16.8 μm in single culture. In contrast, the positive control group with adMSCs had an influence on the neurite length at early time points. After 24 h, the mean length of neurites was significantly increased in co-cultures compared to DRG in single culture (174 ± 80.1/30.2 ± 27.1 μm) and after 48 h 259 ± 50.8/72.9 ± 26.4 μm. (Fig. 5b).

Regarding the number of neurites and number of branching per neuron, no significant differences were observed at both time points in co-cultures with adMSC exosomes compared to the control DRGs, respectively (Fig. 5a, c). The positive control group showed an increased, yet, not statistically significant number of neurites per neuron (Fig. 5c). On the contrary, a significant difference in number of branching per neuron compared to the single DRG cultures (48 h, *P* < 0.001) (Fig. 5a) was presented.

### adMSC Exosome Transplantation for Axonal Regeneration After in Sciatic Nerve Crush Injury

**Fig. 6:**
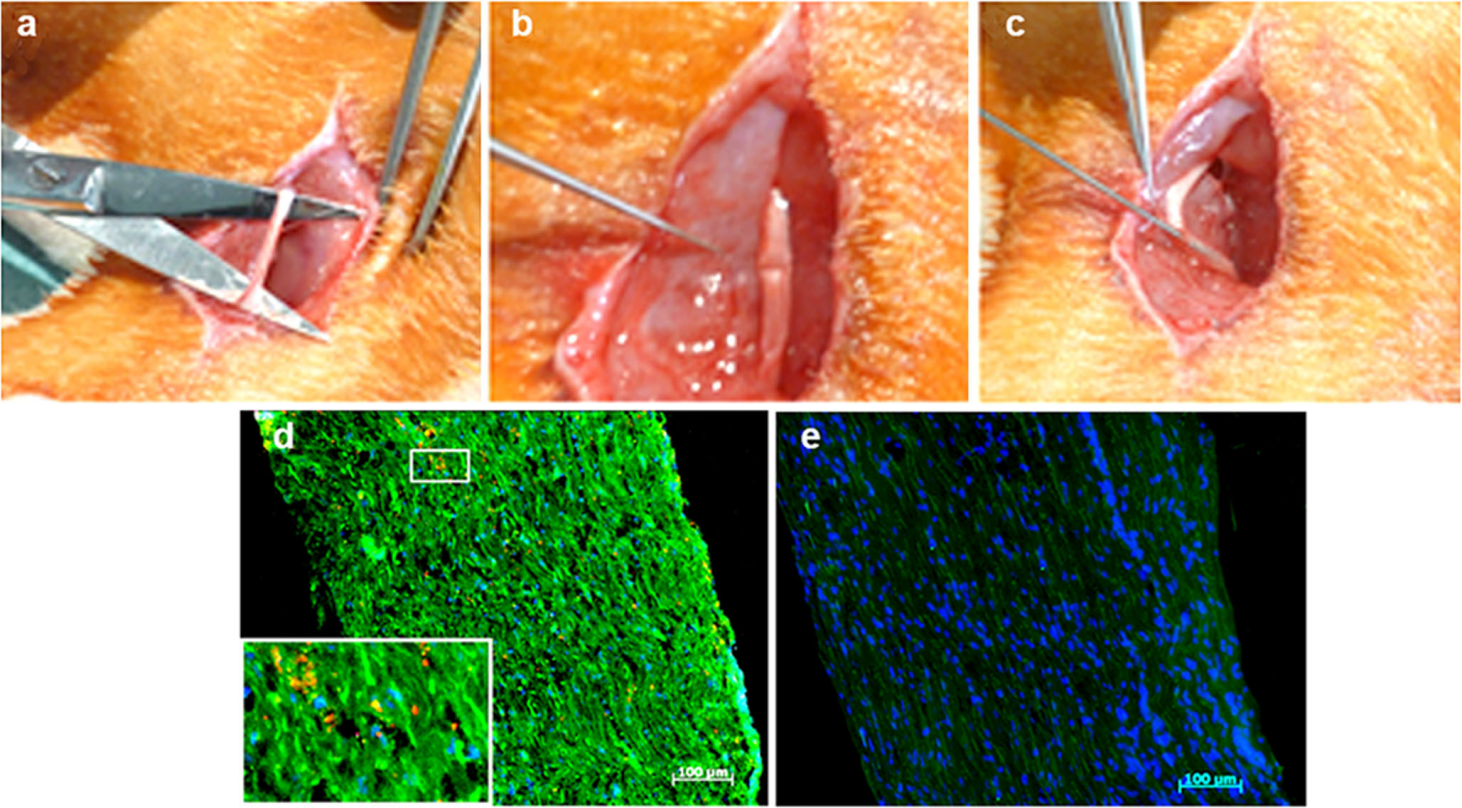
Exosomes implantation. **a**, **b** Sciatic nerve was disposed and crushed with a fine tweezers. **c** The adMSC exosomes were injected proximal and distal. **d** Labelled exosomes in sciatic nerve. Regenerated axons stained with **e** S100 and **f** neurofilament (green). The PKH-26-labelled adMSC exosomes (red)

Next, we evaluated whether adMSC-derived exosomes were internalised in vivo and examined their effect on axonal regeneration after sciatic nerve crush injury as an acute axotomy model. An acute sciatic nerve injury was induced in adult Wistar rats. Proximally and distally from the lesion site, PKH-26-labelled adMSC exosomes were injected (Fig. 6a–c) and integration was evaluated 3 weeks later by immunofluorescent analysis (Fig. 6d). Twenty-one days after implantation, exosome internalisation could be observed in regenerating fibres (Fig. 6d), indicating that adMSC exosomes are internalised by regenerating fibres in vivo and were not phagocytised by macrophages. Regenerated axons in frozen sections of the nerve were stained with neurofilament for determination of axonal regeneration (green). In both experimental groups, there was evidence of increased axonal regeneration and improved functionality. However, the exosome transplantation group (Fig. 6d) had greater numbers of regenerated nerve fibres (ca. 40%) in relation to the neurofilament stained axons in comparison to the control group (Fig. 6e).

### Walking Track Analysis

**Fig. 7:**
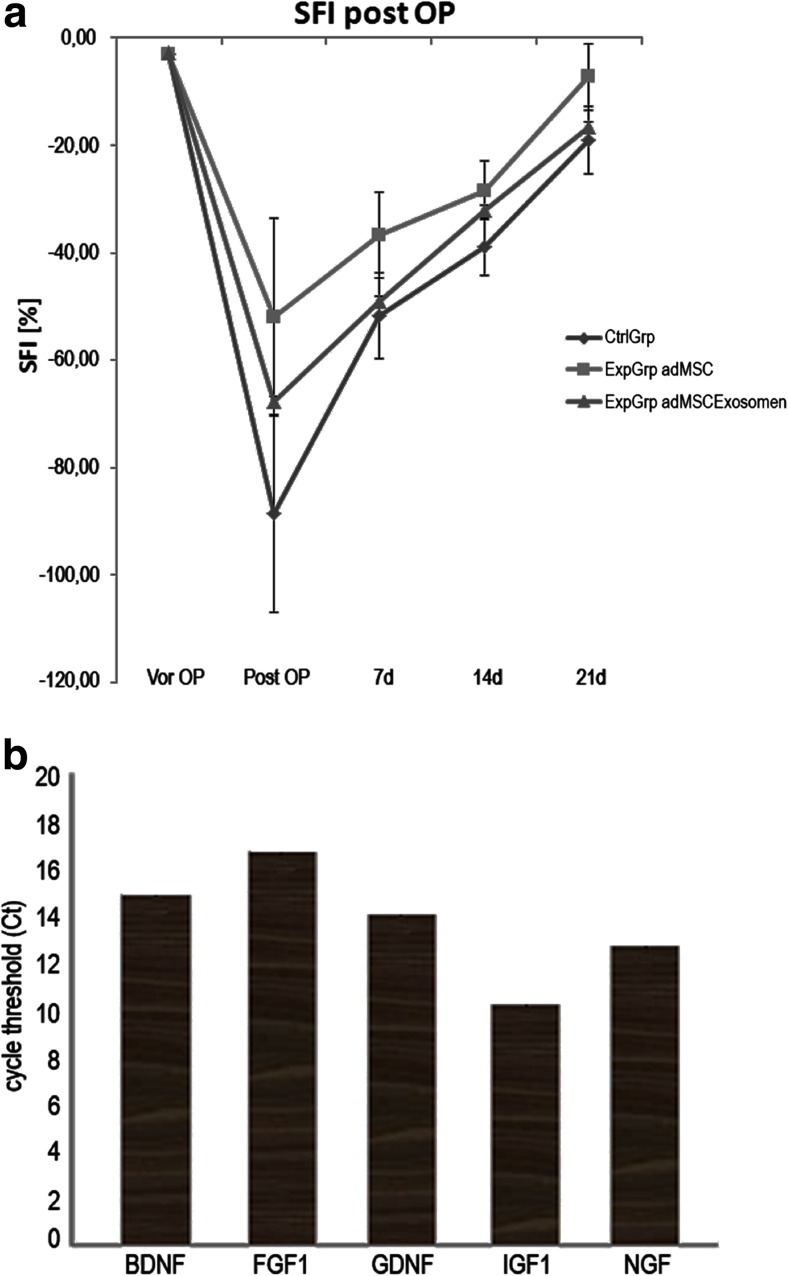
Footprint and growth factors analysis**. a** SFI scores at different time points for the functional recovery from the peripheral injury of the N. ischiadicus. **b** The presence of neural growth factors and mRNA transcripts in adMSC exosomes

To evaluate the efficacy of exosomes on the functional improvement of rats with peripheral nerve injury, the SFI among the two groups, the exosomes group and the negative control group was compared. Typical walking tracks obtained from the negative control group and exosomes groups at 1, 2 and 3 weeks after surgery are presented in Fig. 7a. Preoperative analysis was also performed to determine normal SFI value for each animal. The SFI score is zero for both groups. Immediately following sciatic nerve crush, SFI values dropped to − 88.58 ± 18.4 in control group and − 51.89 ± 18 with adMSCs/− 67.72 ± 46.4 with exosomes experimental groups, respectively, indicating loss of sciatic nerve function. At day 7, the experimental animals group showed greater functional improvement (adMSCs − 36.77 ± 8.0/exosomes − 49.10 ± 27.73) than the sham control groups (0.9% NaCl) (− 51.81 ± 8.01). The injection of adMSC exosomes exerted an improvement (32.20 ± 23.88) in SFI as compared with the control crushed animals (38.98 ± 5.36) starting from 14 days after the injury and ascends at day 21. At day 21 post crush, the exosome-treated animals showed functional improvement (16.75 ± 1.64) compared with control animals group (19.07 ± 6.24). The positive adMSC control group showed an increase but not significant SFI (7.29 ± 6.0) compared to the control group at day 21.

Recent studies have shown that the exosomes derived from stem cells induce biological effects on target tissues by the transfer of genetic material and growth factor proteins. Their small size and flexibility enable them to cross major biological membranes, while their bi-lipid structure protects the RNA and protein cargo from degradation, when facilitating delivery to its target [34]. To identify important genes for the nerve regeneration in the rat adMSC exosomes, we performed gene expression profiling analysis using the PCR array, as described in methods and showed the presence of glial cell-derived neurotrophic factor (GDNF), fibroblast growth factor-1 (FGF-1), brain-derived neurotrophic factor (BDNF), insulin-like growth factor-1 (IGF-1) and NGF transcripts in rat adMSC exosomes for the first time (Fig. 7b). These neurotrophic factors are signalling proteins which support neural survival and axonal growth.

## Discussion

Stem cell-based therapy, with local cell implantation in peripheral nerve injury, has been shown to promote nerve regeneration, with axonal regrowth and myelin formation [24, 42, 44–46]. During this process, stem cells secrete a variety of factors [18, 43], which might positively impact neural cell survival and neuroregeneration. Here, we demonstrate that adMSCs secrete exosomes, which are selectively internalised by axons in vivo, and increase peripheral nerve regeneration after injury. Therefore, transfer of adMSC-derived exosomes to nerve constitutes is a novel mechanism able to facilitate regenerative growth of axons.

Many studies demonstrated the biological effects of exosomes as mediators of intercellular communication. By their ability to transfer proteins, lipids, and RNAs, they can modulate a variety of physiological and pathological processes in the body [47]. The regenerative efficacies of the MSC-derived MVs were observed in a number of recently published studies. MVs have been used to reduce myocardial ischemia/reperfusion injury [32], to reverse fulminant hepatic failures [48] and to protect against acute tubular injury [33]. Here, we could demonstrate the effect on nerve regeneration in vitro and in vivo after characterisation. In this study, the published exosomal markers CD63 and CD9 [49, 50] were detected in these membrane vesicles, further confirming the successful isolation of adMSC exosomes.

We confirmed that the adMSC-derived exosomes were internalised into the Schwann cells. These findings demonstrate that exosomes and their cargo can be transferred between the cells. During the time course, we found that, at 24 h incubation, there were 10% fewer exosomes per cell than at day 14. Twenty-one days later, the number of exosomes per cell decreased again. This may be due to cell division, as there were more cells at 24 h. It is also possible that we have less exosomes per cell, because exosomes that have been taken up early by cells released their cargo into the cytoplasm, and their membrane has either been incorporated into the plasma membrane or degraded [51, 52]. Furthermore, we showed an increased expression of cyclin Ki67, a nuclear protein expressed during G1, S, G2 and M phases of cell cycle and a marker of cell proliferation [53], in Schwann cell’s nuclei, with adMSC exosomes compared to the untreated cells. For the first time, we could demonstrate that the adMSC-derived exosomes stimulate Schwann cell proliferation in vitro.

The primary culture of DRG, either as explants or as cell culture, is an accepted in vitro model for studying peripheral nerve regeneration [54]. Neurite formation and elongation can be examined, e.g. in interaction with other types of cells and/or under special culture conditions [55]. Despite from reducing the numbers of necessary in vivo studies, the in vitro approaches have a lower degree of complexity and are thus easier to define and interpret [56]. Xin et al. has suggested that exosomes might modulate neurite outgrowth in the CNS [33, 57], yet, a direct effect of adMSC exosomes on neurite growth or regeneration has not been described up to now. Our results from cultured DRG neurons treated with adMSC-derived exosomes show the neurite outgrowth of the DRG neurons, determined by increased neurite lengths after 24 h in co-culture. Although the differences were not significant due to the high variances of neurite lengths, there is a tendency for exosomes to enhance neurite outgrowth (Fig. 5). Acceleration of neurite length is of particular interest in peripheral nerve regeneration because often the time period to regenerate successfully is limited.

The role of exosomes has been studied in several areas of neural regeneration [58]. Lopez-Verrilli et al. found that exosomes secreted from Schwann cells increase neurite growth substantially and greatly enhance axonal regeneration in vitro and in vivo [35]. It has furthermore been shown that vesicular-mediated transfer of ribosomes from SCs, the peripheral glial cell type, leads to regenerating axons in vivo [57]. In the present study, adMSC-derived exosomes showed an effect on rat sciatic nerve regeneration by improvement of the nerve regeneration in the exosomes group compared to control group (Fig. 6).

Walking track analysis is a comprehensive test that has been used widely for evaluating the recovery of motor function as a result of post traumatic regeneration of peripheral nerve in rats [59]. The results of the present study showed that the adMSC exosomes, compared to the control group, have a faster improvement in the functional recovery of the sciatic nerve in the course of time (Fig. 7a).

The beneficial effect of adMSCs transplantation in regeneration of peripheral nerve injuries has been shown in several studies [60, 61]. Most of the beneficial effects exerted by the MSC are strongly correlated with the production of neurotrophic substances, such as FGF, NGF, ciliary neurotrophic factor, BDNF and GDNF [62–64]. It is known that the microvesicles act as shuttles for selective pattern of enzymes, cytokines and trophic molecules at both protein and messenger RNA levels. Their attachment or fusion in peripheral cell membrane may cause cell regeneration or genetic reprogramming, inhibition of inflammation and enhancement of angiogenesis [33, 65]. Reportedly, MVs participate in myelin formation [66], in neurite outgrowth and neuronal survival [67]. MVs can transfer bioactive molecules and deposit packaged bioactive effectors such as specific genes, small organelles or a cocktail of cytokines from MSCs to the injured tissue [32]. Our group demonstrated the presence of growth factors, BDNF, FGF-1, GDNF, IGF-1 and NGF in the adMSC exosomes for the first time (Fig. 7b). This result could represent one mean by which these exosomes act on the enhancement of axon regeneration in vivo.

In conclusion, with the expansion of the number of published studies on exosomes in the past years, it is clear that research on secreted exosomes and their role as intercellular messengers is an exciting field. The results of the present study indicate that this novel nerve repair function of adMSC-derived exosomes could potentially engender new approaches of nerve regeneration research.
